# PET imaging of mitochondrial function in acute doxorubicin-induced cardiotoxicity: a proof-of-principle study

**DOI:** 10.1038/s41598-022-10004-6

**Published:** 2022-04-12

**Authors:** Felicitas J. Detmer, Nathaniel M. Alpert, Sung-Hyun Moon, Maeva Dhaynaut, J. Luis Guerrero, Nicolas J. Guehl, Fangxu Xing, Pedro Brugarolas, Timothy M. Shoup, Marc D. Normandin, Matthieu Pelletier-Galarneau, Georges El Fakhri, Yoann Petibon

**Affiliations:** 1 Gordon Center for Medical Imaging, Massachusetts General Hospital, Harvard Medical School, 125 Nashua St. Suite 660, Boston, MA 02114-1101 USA; 2 Surgical Cardiovascular Laboratory, Massachusetts General Hospital, Harvard Medical School, Boston, MA USA

**Keywords:** Preclinical research, Cardiology

## Abstract

Mitochondrial dysfunction plays a key role in doxorubicin-induced cardiotoxicity (DIC). In this proof-of-principle study, we investigated whether PET mapping of cardiac membrane potential, an indicator of mitochondrial function, could detect an acute cardiotoxic effect of doxorubicin (DOX) in a large animal model. Eight Yucatan pigs were imaged dynamically with [^18^F](4-Fluorophenyl)triphenylphosphonium ([^18^F]FTPP^+^) PET/CT. Our experimental protocol included a control saline infusion into the left anterior descending coronary artery (LAD) followed by a DOX test infusion of either 1 mg/kg or 2 mg/kg during PET. We measured the change in total cardiac membrane potential (ΔΨ_T_), a proxy for the mitochondrial membrane potential, ΔΨ_m_, after the saline and DOX infusions. We observed a partial depolarization of the mitochondria following the DOX infusions, which occurred only in myocardial areas distal to the intracoronary catheter, thereby demonstrating a direct association between the exposure of the mitochondria to DOX and a change in ΔΨ_T_. Furthermore, doubling the DOX dose caused a more severe depolarization of myocardium in the LAD territory distal to the infusion catheter. In conclusion, [^18^F]FTPP^+^ PET-based ΔΨ_T_ mapping can measure partial depolarization of myocardial mitochondria following intracoronary DOX infusion in a large animal model.

## Introduction

Doxorubicin (DOX) is a widely used chemotherapeutic for treatment of a variety of cancers, either as a primary intervention or in combined therapy. While DOX therapy often results in increased cancer survival, it can also be accompanied by cardiotoxic effects, resulting in myocardial injury. Current methods for detection of cardiotoxicity rely on serial evaluation of a patient’s left ventricular ejection fraction (LVEF). However, LVEF decline is a lagging index of cardiotoxic injury^1,2^ and by the time left ventricular function is impaired, damage is often irreversible. Therefore, new imaging approaches for detecting subclinical cardiotoxicity are greatly needed^3,4^.

The mechanisms underlying doxorubicin-induced cardiotoxicity (DIC) are complex and incompletely understood. Nevertheless, a key role has been attributed to mitochondrial damage and dysfunction^5^. Mitochondria are indeed the most extensively injured intracellular organelles upon exposure to DOX and mitochondrial injury is believed to represent one of the first events of cardiotoxicity^5,6^. Hence, monitoring cardiac mitochondrial function might allow early detection of DIC^7,8^.

The mitochondrial membrane potential (ΔΨ_m_) is a comprehensive index of mitochondrial function. It provides the energy for mitochondrial adenosine triphosphate (ATP) production via oxidative phosphorylation, which is essential for normal cardiac function. In normal physiologic function, ΔΨ_m_ lies within a narrow range; dysregulation of ΔΨ_m_ results in increased reactive oxygen species production, leading to oxidative damage to DNA, proteins, and lipids, mitochondrial dysfunction, and ultimately cell death^9,10^. Importantly, most known mechanisms underpinning mitochondrial dysfunction in DIC, particularly inhibition of the electron transport chain, are associated with depolarization of ΔΨ_m_. Notably, depolarization of ΔΨ_m_ following exposure to DOX has been observed in-vitro in various cell lines, such as cardiomyocytes^11^ and carcinoma cells^12^ as well as ex-vivo in Langendorff-perfused rat hearts^13^.

Lipophilic cations like tritiated tetraphenylphosphonium (TPP^+^) have been used for decades to measure ΔΨ_m_ in-vitro^14^. More recently, lipophilic cationic PET tracers, such as [^18^F](4-Fluorophenyl)triphenylphosphonium ([^18^F]FTPP^+^)^15,16^, [^18^F]fluorobenzyl-triphenylphosphonium^17^ and [^18^F]MitoPhos^7^ have been introduced, enabling *in-vivo* assessment of ΔΨ_m_. Our group has recently developed an approach to quantify cardiac tissue membrane potential (ΔΨ_T_), a proxy of ΔΨ_m_, in units of millivolts with [^18^F]FTPP^+^ PET^16^. We have further shown that cardiac ΔΨ_T_ is sensitive to changes induced by a proton gradient uncoupler causing partial depolarization of ΔΨ_m_^18^. We have also demonstrated that ΔΨ_T_ mapping with [^18^F]FTPP^+^ PET is feasible in human subjects, resulting in measured values in excellent agreement with those in the literature^19^.

Given the established role of mitochondrial dysfunction in DIC, we hypothesized that PET-based ΔΨ_T_ mapping could provide an early biomarker of DIC. As a first step in testing this hypothesis, we evaluated whether our approach could detect a cardiotoxic effect of DOX in an acute setting. In this proof-of-principle study, we used a porcine model in which DOX was infused into the left anterior descending (LAD) coronary artery during [^18^F]FTPP^+^ PET imaging. Through this localized administration, the experiments were designed such that ΔΨ_T_ could be measured before and during intracoronary infusion of DOX. While the DOX administration was thus different from a clinical IV infusion, with our protocol, each animal served as its own control, thereby allowing more sensitive detection of mitochondrial depolarization.

## Materials and methods

Eleven male Yucatan pigs (45–56 kg, 9–11 months, Sinclair Bio-Resources, Columbia, MO), of which three were later excluded from data analysis as explained below, were studied. All animals were housed and maintained under the supervision of the veterinary staff of the Center for Comparative Medicine at Massachusetts General Hospital (MGH). Experiments were performed in accordance with the US. Department of Agriculture (USDA) Animal Welfare Act and Animal Welfare Regulations (Animal Care Blue Book), Code of Federal Regulations (CFR), Title 9, Chapter 1, Subchapter A, Part 2, Subpart C, §2.31. 2017. The study protocol was approved by the MGH Institutional Animal Care and Use Committee in accordance with The Guide for the Care and Use of Laboratory Animals^20^ and all methods were carried out following relevant guidelines and regulations. For this proof-of-principle study, a small sample size was used and no blinding was performed for the data analysis. The methods are reported in accordance with ARRIVE guidelines^21^.

### Animal preparation

The animals were premedicated for three days with amiodarone (150–200 mg) prior to the study, fasted overnight, and anesthetized via intramuscular administration of a mixture of Telazol (4.4. mg/kg) and Xylazine (2.2 mg/kg). Anesthesia was maintained during the entire experiment with a mixture of isoflurane (1.25–3%) and oxygen. A constant infusion of amiodarone (50–150 mg over 2–3 h) was administered during the surgery.

For [^18^F]FTPP^+^ injection, a 22G catheter was placed into an auricular vein. To administer fluids, medications, and CT contrast, another 4–6 Fr catheter was inserted into a femoral vein, and a femoral artery was cannulated for arterial blood sampling. A left thoracotomy was performed via a skin incision; the pericardium was opened, and a side branch of the mid left anterior descending artery (LAD) was isolated. A 22G angiocatheter was inserted into the lumen of the LAD via the side branch, then exteriorized through the chest wall, and secured on the skin for saline/DOX infusions.

### Experimental protocol

**Figure 1:**
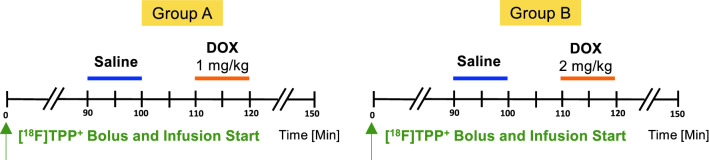
Timeline of saline and DOX infusions during the PET image acquisition for Group A and B.

Figure 1 provides an overview of the experimental protocol. The animals were divided into two groups, A and B, as discussed below. Each animal underwent dynamic [^18^F]FTPP^+^ PET imaging for up to three hours on a hybrid PET/CT (GE Discovery MI) using a bolus-plus-infusion protocol^22^. When the tracer concentrations reached secular equilibrium—typically after 90 min–0.5 ml/kg of saline was infused into the LAD over 10 min to serve as a negative control. Ten minutes after the end of the saline infusion, a DOX infusion (doxorubicin hydrochloride, Millipore Sigma, Saint Louis, MO) was likewise administered over 10 min into the LAD. Group A (resp. B) included 5 (resp. 6) animals, each of which received a DOX infusion of 1 mg/kg (resp. 2 mg/kg) dissolved in 0.5 ml/kg saline. At the end of the procedures, the animals were euthanized with 100 mg/kg Euthasol (Virbac Animal Health, Westlake, TX) administered IV consistent with AVMA guidelines^23^.

Three animals were excluded from the study due to technical problems during surgical preparation and/or difficulties with LAD catheter placement, resulting in four animals in Group A (P1, …, P4) and four animals for Group B (P5, …, P8), for a total of eight pigs.

### Quantification of myocardial membrane potential

The kinetic and biophysical basis of our PET-based membrane potential mapping technique has been detailed elsewhere^16,24^. Briefly, the total myocardial membrane potential ΔΨ_T_, which represents the sum of the cellular and mitochondrial membrane potentials, can be approximated as $\Delta {\Psi }_{T}\approx \frac{1}{\beta }ln\left[\frac{\left(1-{f}_{ECV}\right){\cdot f}_{mito}}{{V}_{T}}\right]$ ^16^, where β is a physical constant and *f*_mito_ and *f*_ECV_ refer to the mitochondrial and extracellular volume fractions. The total volume of distribution, V_T_, can be measured at secular equilibrium by taking the ratio of the tracer concentration in tissue over that in plasma. The value of *f*_mito_ was set to 0.26^16,25^. The extracellular volume fraction, *f*_ECV_, was calculated based on pre- and late post-contrast CT images as outlined below.

### PET/CT imaging and blood sampling

[^18^F]FTPP^+^ (molar activity 90.5 ± 38.1 GBq/μmol end of synthesis) was synthesized and purified on a GE Tracerlab FX2N synthesis unit using [^18^F]fluoride produced onsite by a GE PETtrace cyclotron bombarding > 98% enriched [^18^O]water as previously reported^15^. [^18^F]FTPP^+^ was formulated in sterile saline for injection (0.9% sodium chloride solution) and EtOH (6% bolus; 0.8% infusion). PET data were acquired dynamically in list mode for up to three hours beginning from bolus administration of 601.1 ± 83.9 MBq [^18^F]FTPP^+^ and start of an infusion of 183.1 ± 27.3 MBq over 180 min (3 mL/min). Arterial blood samples were manually drawn every five minutes, starting from the beginning of the saline infusion.. Tracer concentration in plasma was measured with a calibrated well counter and was expressed as Bq/cc. Following PET, diagnostic-quality CT images were acquired for quantification of myocardial *f*_ECV_^26^. First, a pre-contrast scan was acquired. A post-contrast image was acquired ten to fifteen minutes after an IV contrast injection (1.8 mL/kg Iodine 300 (Ultravist, Bayer Healthcare, Pittsburgh, PA) or 1.45 mL/kg Iodine 370 (Bracco Diagnostic, Monroe Township, NJ)) into the femoral vein using a power injector. Pre- and post-contrast images had a voxel size of 0.65 × 0.65 × 2.5 mm^3^ and were acquired with a tube voltage of 100–120 kVp. Details of the imaging parameters for each animal are shown in Suppl. Table 1.

### Data processing

List mode PET data were reconstructed dynamically (frames: 12 × 5, 6 × 10, 6 × 30, and 175 × 60 s) using OSEM with CT-based attenuation correction, resulting in radioactivity concentration maps in units of Bq/cc, with 89 slices and a voxel size of 2.73 × 2.73 × 2.8 mm^3^. The dynamic PET images were transformed into short axis. Next, defined frames of the experiment (see below) were summed and non-rigidly registered using ITK^27^ to an in-house developed cardiac [^18^F]FTPP^+^ atlas constructed using data acquired in four healthy pigs. The inverse deformation fields were then applied to the masks of the standard 17 myocardial segments^28^, which had been defined in atlas space, to obtain subject-specific masks for all segments. Time activity curves (TACs) for the 17 segments were extracted for each animal from the average segmental concentration for each frame.

*f*_ECV_ was calculated based on the pre- and post-contrast CT images^26^ and the average value over the entire myocardium (excluding the apex, segment 17) was used for subsequent quantification of ΔΨ_T_. V_T_ before and after the saline and DOX infusions was calculated from the average [^18^F]FTPP^+^ concentration in tissue and arterial plasma for the different phases of the experiment, as defined in Suppl. Table 2. Different data analysis periods were used to calculate the average tracer concentration before and after the 1 and 2 mg/kg DOX infusions because of apparent differences in their temporal responses. The tracer concentration in plasma was assumed to be constant starting from the time of the saline infusion and was calculated as the average concentration over time. Next, ΔΨ_T_ was quantified based on V_T_ for each phase using the equation introduced above.

Parametric images of ΔΨ_T_ were also computed from the dynamic PET images for the different phases of the experiment using the same approach.

To help visualize experimental results, we present segmental concentrations averaged over the cohorts of pigs for all measurement times. Tissue tracer concentrations were normalized by animal weight and the cumulative tracer activity injected by bolus-plus-infusion until time t, i.e., $TAC_{{norm}} (t) = \frac{{TAC(t)}}{{\int_{0}^{t} {C(u)_{{inj}} du/W} }}$, where ${\int }_{0}^{t}C{(u)}_{inj}du$ refers to the administrated tracer activity up to time t (MBq) and *W* is the animal weight (g). This normalization is an extension of the well-known standard uptake value (SUV) index, taking time dependence into account.

### Statistical analysis

For the purpose of hypothesis testing we used the change in ΔΨ_T_ (δΔΨ_T_) for each of the infusions (e.g. δΔΨ_T,saline_ = ΔΨ_T,after_saline_—ΔΨ_T,before_saline_) as the dependent variable in a mixed effects statistical model with “myocardial segment” (1,2, …,16) and “treatment type” (saline vs. DOX of 1 mg/kg vs. DOX of 2 mg/kg) as categorical variables. We first tested whether a model with “treatment type” and “myocardial segment” was able to explain the observed data (ANOVA omnibus test). Next, we specifically tested three hypotheses: whether δΔΨ_T_ was significantly different (1) for segments 7, 8, 13, and 14 (all part of the LAD territory) compared to all other segments, (2) for the saline infusion compared to the DOX infusion, and (3) for the DOX infusion with a dose of 1 mg/kg compared to the 2 mg/kg infusion. These a-priori hypotheses were tested as planned contrasts within a linear mixed effects model, taking account of repeated measures in the same animal.

Due to the localized administration of the infusions, the effects of DOX and saline were expected to differ between segments. Therefore, we also included two interaction terms between the contrasts of hypothesis (1) and (2) and between (1) and (3), to test whether the change in ΔΨ_T_ was significantly different between the saline and DOX infusion as well as for the different DOX doses when taking differences between segments (i.e., LAD vs. non-LAD segments) into account.

The tested contrasts were considered significant for *p* < 0.05. Segment 17 was excluded from the analysis due to difficulties in accurate segmentation of the apex.

All statistical analyses were performed in R^29^, the linear mixed effects model was applied using the lme4 package^30^. *P*-values from approximate F- and t-tests were calculated using Satterthwaite’s method for approximating the degrees of freedom^31^, implemented in lme4.

## Results

**Figure 2:**
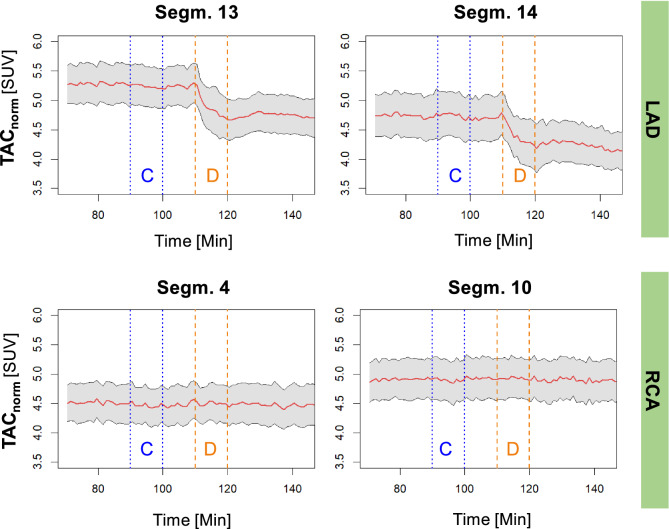
Mean normalized TACs for two segments of the LAD (top) and two segments of the RCA (bottom) for the eight animals. The grey shaded area indicates the mean + /-standard error. The blue dotted lines indicate the beginning and end of the control saline infusion (C), whereas the orange dotted lines mark the beginning and end of the DOX infusion (D).

Figure 2 provides an empirical summary of the results of the experiment, showing the mean normalized tracer concentrations in tissue over time for the eight animals. For segments of the LAD territory (e.g., 13 and 14, Fig. 2, top), a clearly visualized decrease in [^18^F]FTPP^+^ concentration occurred, starting from the beginning of the DOX infusion. No such change was observed for the control segments (e.g., RCA segments 4 and 10, Fig. 2, bottom), nor for the control saline infusion. This localized reduction in tracer concentration signifies partial mitochondrial depolarization and clearly indicates an effect due to DOX that may be appreciated without a formal statistical analysis. Representative examples of normalized TACs for group A and B are shown in Suppl. Figure S1. For group A (1 mg/kg DOX infusion), the tissue tracer concentration tended to recover after the DOX infusion to a value close to the baseline value, whereas for group B (2 mg/kg DOX infusion), the observed effect was generally less reversible.

**Figure 3:**
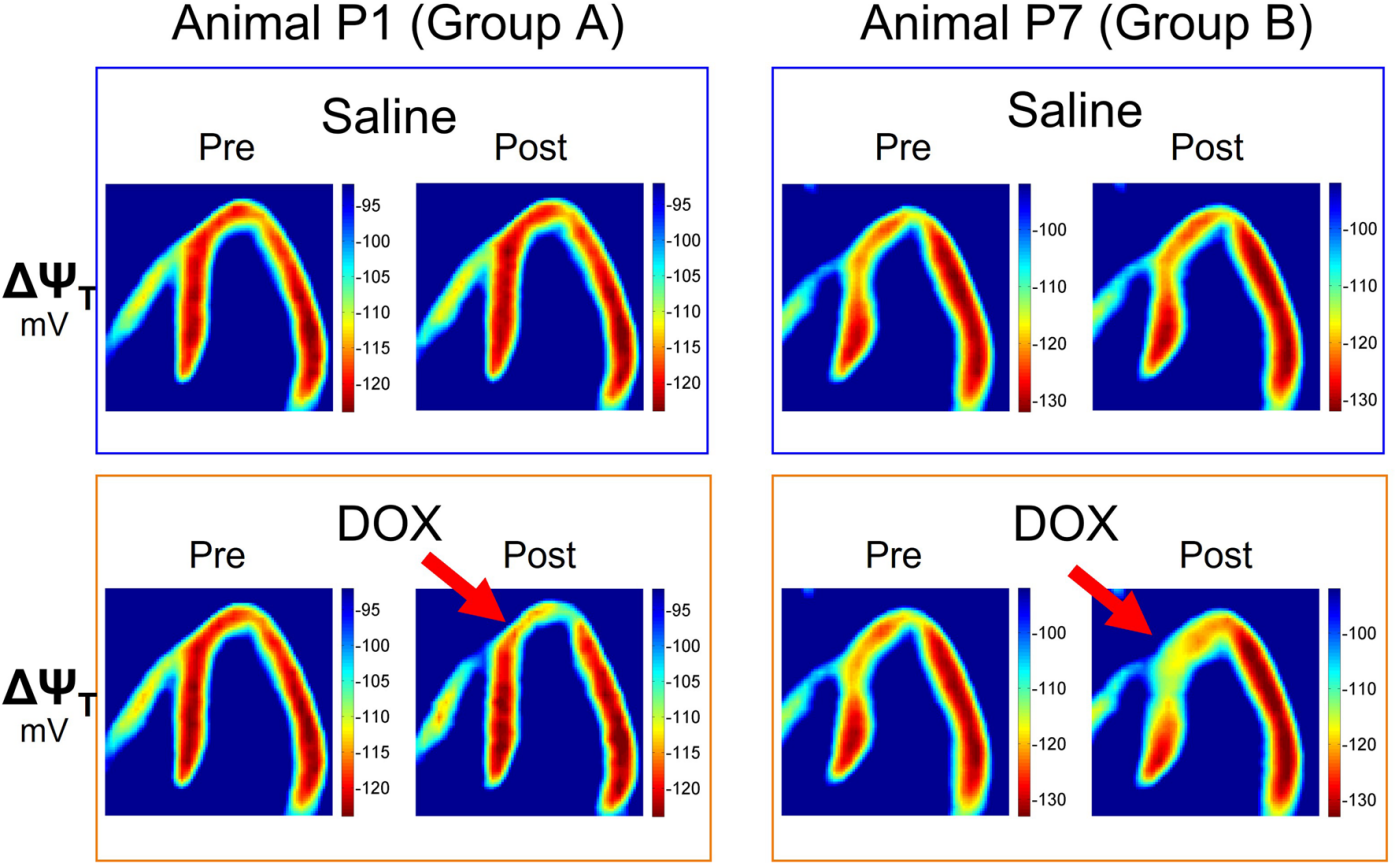
Parametric images of ΔΨ_T_ before and after the saline (top) as well as the DOX infusion (bottom) for one animal of group A (left) and one animal of group B (right).

The localized reduction in myocardial [^18^F]FTPP^+^ concentration can also be appreciated on the parametric images of the apparent ΔΨ_T_ presented for two example animals in Fig. 3 and Suppl. Figs. S2 and S3. They show clear evidence for a partial depolarization of ΔΨ_T_ after the DOX infusion in the apical septal area of the myocardium, i.e., the area distal to the LAD catheter, but not after the saline infusion. Overall, a partial depolarization of ΔΨ_T_ after the DOX infusion was observed for seven of the eight animals. For those animals, the maximum change in apparent ΔΨ_T_ over all segments ranged between 2.5 mV (P6) and 10.5 mV (P3, see Table 1). For all animals, the maximum ΔΨ_T_ change occurred in the LAD territory, particularly segment 13 or 14, i.e., the apical anterior or apical septal area of the myocardium.

**Table 1:** Maximum change in ΔΨ_T_ for segment with largest depolarization for each studied animal.

Animal	Group	Segment	δΔΨ_T_ [mV]
P1	A	14	3.212
P2	A	13	4.478
P3	A	13	10.549
P4	A	13	0.513
P5	B	14	5.107
P6	B	14	2.504
P7	B	14	6.510
P8	B	14	5.457

**Figure 4:**
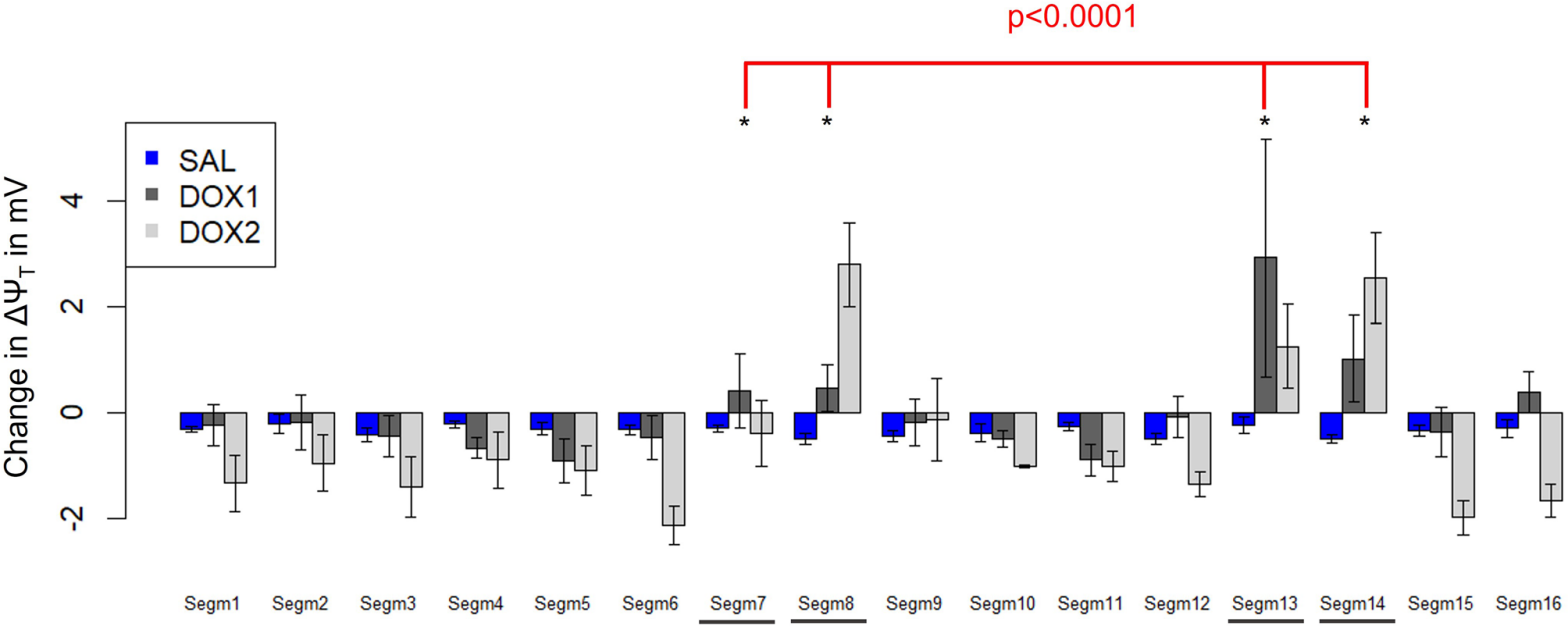
Mean (bars) + /- standard error of change in ΔΨ_T_ for each of the 16 segments and the three types of “interventions” (saline – “SAL”, DOX 1 mg/kg – “DOX1”, and DOX 2 mg/kg – “DOX2”) averaged over the eight studies. The four segments that had a significantly different change in ΔΨ_T_ compared to the other segments are indicated with asterisks.

**Figure 5:**
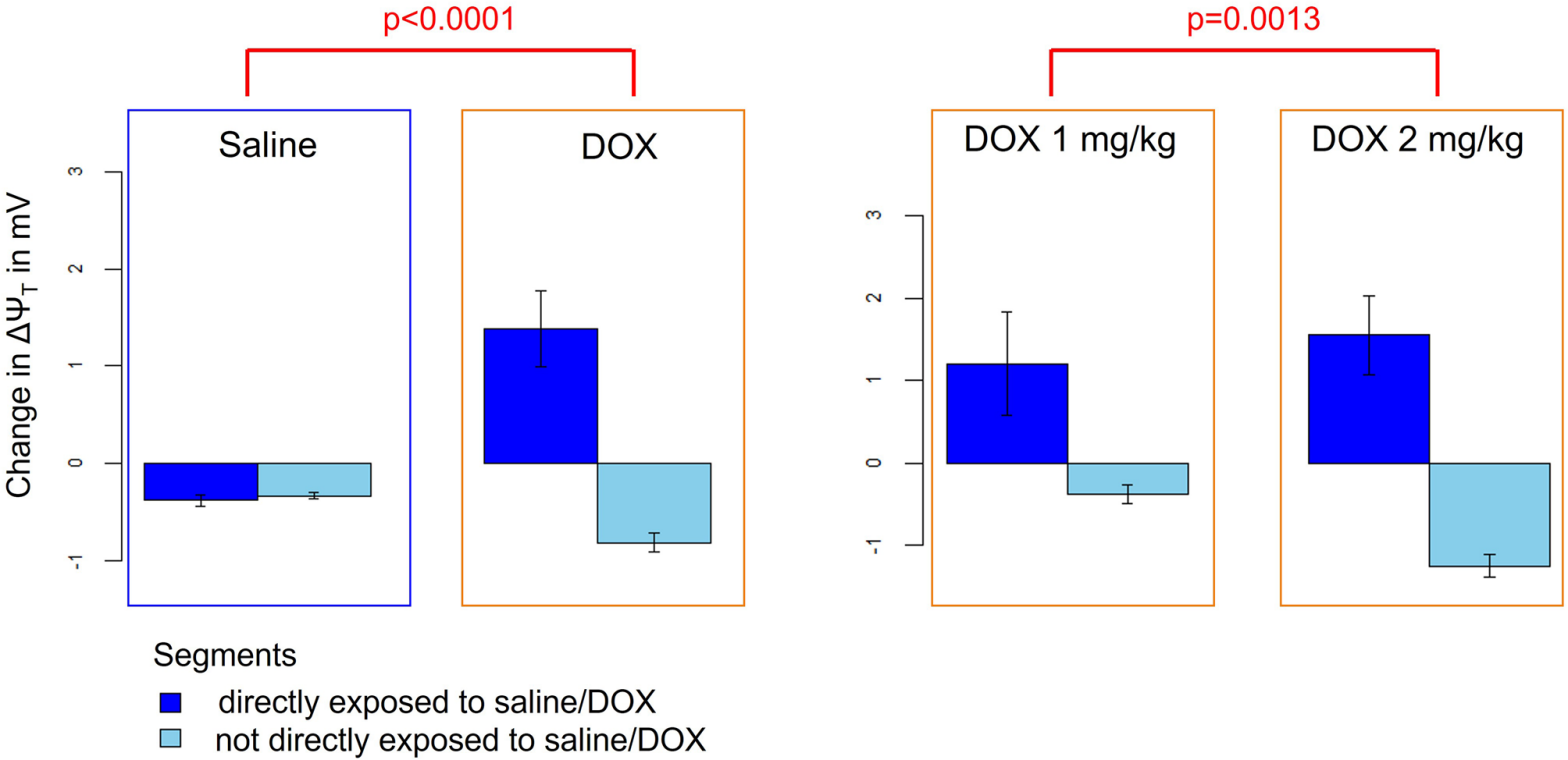
Mean (bars) + /- standard error of change in ΔΨ_T_ for the segments directly exposed to the saline and DOX infusions (a priori defined as segments 7, 8, 13, and 14) as well as the control segments after the saline and DOX infusion (left) and comparison of change in ΔΨ_T_ for a dose of 1 mg/kg vs 2 mg/kg (right).

The results of the statistical analysis are summarized in Figs. 4 and 5 as well as Suppl. Table S3. The change in ΔΨ_T_ was significantly associated with the categorical variables “segment” and ”treatment type” (*p* < 0.0001 and *p* = 0.04, respectively). More specifically, δΔΨ_T_ was significantly different for the segments directly exposed to DOX (a-priori defined as segments 7, 8, 13, and 14) compared to the other segments (*p* < 0.0001). The change in ΔΨ_T_ after the DOX infusion was significantly different from the change after the control saline infusion when taking different effects for LAD vs. non-LAD segments into account (*p* < 0.0001): For the segments directly exposed to DOX, ΔΨ_T_ was partially depolarized. Much smaller changes in ΔΨ_T_—often in the opposite direction (i.e., more negative ΔΨ_T_)—occurred for the other segments as well as for the control saline infusion. Furthermore, δΔΨ_T_ was significantly larger for a DOX infusion of 2 mg/kg compared to 1 mg/kg (*p* = 0.0013).

The more negative ΔΨ_T_ observed after the DOX infusion for some of the segments not part of the LAD territory (which can be seen in Figs. 4 and 5) can be explained by the fact that perfect secular equilibrium was not reached in all the experiments. Therefore, the [^18^F]FTPP^+^ concentration in tissue continued to increase moderately during the data acquisition for those segments not affected by the DOX infusions, resulting in a more negative apparent ΔΨ_T_.

Overall, these results demonstrate that DOX infusion caused a significant change in ΔΨ_T_, indicating a partial depolarization of cardiac mitochondria. These changes occurred only in myocardial areas distal to the intracoronary catheter, demonstrating a causal association between the direct exposure of the mitochondria to DOX and partial depolarization of ΔΨ_T_ as measured using our proposed technique. Furthermore, doubling DOX dose caused a more severe depolarization of the myocardial mitochondria.

## Discussion

This study is the first to non-invasively measure a partial depolarization of myocardial mitochondria after acute exposure to DOX in a large animal model. Mitochondrial damage plays a central role in DIC with direct effects of DOX on mitochondrial function^5^. Consequently, imaging of mitochondrial function may enable early detection of DIC. Voltage sensitive tracers such as [^18^F]FTPP^+^ accumulate in the mitochondria according to the Nernst equation, relating the tracer concentration on each side of the inner mitochondrial membrane to ΔΨ_m_, and thus enabling imaging of mitochondrial function. We have previously developed an approach for quantifying ΔΨ_T_, a proxy of ΔΨ_m_, in mV with [^18^F]FTPP^+^ PET^16,19^. The current study extends these results, clearly establishing that this technique can measure a depolarization of myocardial mitochondria following an acute DOX challenge in-vivo in a porcine model. While DOX-induced cardiotoxicity often manifests after several months of treatment^5^, this proof-of-principle study is an important first step providing the rationale for more detailed studies mimicking clinical therapeutic protocols.

New approaches for early diagnosis of cardiotoxicity are greatly needed^3^. Methods under investigation include echocardiography-based assessment of LV global longitudinal strain (GLS)^32,33^ and cardiac MRI for measurement of *f*_ECV_^34^ or T2 mapping for detection of edema^35^. While GLS decline was associated with a later drop of LVEF^32,36^, a recent prospective randomized study did not identify a significant benefit of GLS-guidance over LVEF-based strategy for initiation of cardioprotective therapy^33^. This finding suggests a need for even earlier detection of cardiotoxicity. Similarly, cardiac MRI-based assessment relies on initial myocardial damage to detect an increase in *f*_ECV_ or edema^37^.

With our imaging approach, we were able to measure a localized decrease of myocardial [^18^F]FTPP^+^ concentration following acute exposure to DOX. An important strength of our study design pertains to the intracoronary infusion of DOX, directly into the LAD, resulting in exposure of specific segments of the myocardium to this drug. That is, we only observed a significant change in ΔΨ_T_ for LAD segments distal to the intracoronary catheter. This setting allows us to relate the observed drop in myocardial tracer concentration directly to the exposure of the corresponding segments to DOX. We observed an immediate effect of DOX on cardiac membrane potential. This aspect is an important difference compared to LVEF measurement, where a change can only be identified once—often irreversible—damage to the myocardium has occurred. Furthermore, the maximum change in ΔΨ_T_ was (for the studies with a detectable effect of DOX) in the range of 2.5–10.5 mV; for none of the studies was ΔΨ_T_ fully depolarized. Based on this observation, we speculate that DOX injury may affect subsets of mitochondria differently, resulting overall in partial depolarization of ΔΨ_T_ for a segment: some mitochondria might not be affected by DOX and remain fully functional, others might recover function after initial injury, and a third group of mitochondria might become completely dysfunctional. We expect the number of mitochondria falling into the last group to increase over the course of chemotherapy. In other words, mitochondrial damage—measurable in terms of drop in ΔΨ_T_—may add up as the cumulative DOX dose increases, allowing, when detected early, for implementation of cardioprotective interventions before ultimate mitochondrial dysfunction and irreversible myocardial damage.

We measured ΔΨ_T_, the sum of the cellular membrane potential (ΔΨ_c_) and ΔΨ_m_, as a proxy of ΔΨ_m_. ΔΨ_c_, which represents the average cellular membrane potential over a cardiac cycle, is correlated with the heart rate. For our studies, we observed a largely constant heart rate during and after the DOX infusions. However, DOX is known to affect ion currents across the cellular membrane, thus, potentially altering ΔΨ_c_^38^. Nevertheless, ΔΨ_m_ represents the major part of ΔΨ_T_ so that the observed changes in ΔΨ_T_ are most likely related to a partial depolarization of ΔΨ_m_.

### Study design and comparison to clinical treatment with DOX

For our study, we administered between 1 and 2 mg/kg of DOX, which is comparable to the dose given clinically to treat patients^39^. However, there is an important difference. In our study, the entire dose of 1–2 mg/kg of DOX was delivered directly to the myocardium, and more specifically the vascular territory perfused by the LAD. The direct infusion of DOX into the LAD cannot be directly translated to human investigation; in clinical treatment DOX is administered IV and thus permeates the entire myocardium, as well as other organs. Using the reported area under the curve of 4.2 μmol*hour/L for an IV infusion of 1.62 mg/kg of DOX over 15 minutes^40^, we computed the estimated exposure of the LAD territory from an IV administration of DOX as 0.7647 mg * hour. In contrast, assuming no clearance during the intracoronary DOX infusion as well as full clearance after the end of the infusion, the LAD territory was exposed to 4.167 mg * hour of DOX for an intracoronary infusion of 1 mg/kg. Based on this estimate, the DOX exposure in our setting was approximately five times higher compared to an IV infusion. This finding indicates a potentially smaller change of ΔΨ_T_ after one cycle of clinical DOX treatment than observed in our study. At the same time, as described above, the partial depolarization of ΔΨ_T_ is likely to increase over several cycles of DOX administration. Identifying a threshold of depolarization in ΔΨ_T_ for definition of subclinical cardiotoxicity will require clinical studies. Moreover, the partial depolarization of ΔΨ_T_ that we observed likely resulted from acute cardiotoxic effects of DOX, such as DOX binding to cardiolipin, a phospholipid present in the mitochondrial membrane involved in electron transport chain activity and thus required for mitochondrial ATP production^5,41,42^. On the other hand, chronic effects of DOX involve binding to topoisomerase 2β, which is largely present in mitochondria, resulting in DNA double-strand breaks and transcriptome changes leading to defective mitochondrial biogenesis and formation of reactive oxygen species^5,43–45^. The relation between the immediate change in ΔΨ_T_ that was observed in our acute study and more chronic cardiotoxicity will need to be elucidated in further studies.

In our study, anesthesia was initiated with a mixture of Telazol and Xylazine and maintained with isoflurane. Isoflurane has a known depolarizing effect on ΔΨ_m_ and the observed magnitude of change in ΔΨ_T_ could also be influenced by isoflurane^46^. However_,_ it is unlikely that the observed changes in ΔΨ_T_ were caused by anesthesia since isoflurane levels, which were monitored with a gas analyzer, remained constant during imaging. Furthermore, the combination of Xylazine with Telazol/Zolazepam has been associated in pigs with cardiovascular effects, such as a lowered heart rate^47^ so that a similar effect might be caused by Xylazine-Telazol. Hence, these two drugs could have affected the baseline cellular membrane potential ΔΨ_c_ and thus ΔΨ_T_, but unlikely influenced the observed change in ΔΨ_T._

### Effect of different DOX doses

For the higher dose of 2 mg/kg, a significantly different change in ΔΨ_T_ compared to the 1 mg/kg dose occurred. We further observed that for a dose of 1 mg/kg, the DOX infusion often had a transient effect on the [^18^F]FTPP^+^ concentration in the affected segments, whereas, for the higher dose, the concentration did not recover to its value prior to the infusion (see Suppl. Fig. S1 for example TACs). While the mechanisms behind these different effects are not clear, we speculate that some mitochondria can recover from initial, temporary exposure to DOX for lower doses, whereas this ability is limited for higher doses. Furthermore, the maximum decrease in ΔΨ_T_ overall occurred later when administering a dose of 2 mg/kg. For this reason, we computed the change in ΔΨ_T_ for a dose of 2 mg/kg based on different frames compared to the 1 mg/kg dose.

### Other approaches for imaging of mitochondrial function

Other groups have recently evaluated the use of other lipophilic cations for PET imaging such as [^18^F]MitoPhos^7^ or [^68^ Ga]Galmydar^48^ to detect signs of cardiotoxicity in an acute setting, where rodents received DOX intravenously 2–5 days before PET imaging. Compared to the control animals, a significantly lower myocardial tracer uptake was observed, indicating a decrease in ΔΨ_m_; however, absolute quantification of membrane potential was not performed. Furthermore, unlike [^18^F]FTPP^+^, these tracers have not yet been translated to humans. SPECT imaging with 99mTc-sestamibi, a routinely used cardiac perfusion tracer whose distribution in myocardium is based both on perfusion and ΔΨ_m_, has also been proposed for detection of DIC^49^. Besides the poorer spatial resolution and quantification of SPECT compared to PET, the dependence of 99mTc-sestamibi distribution in myocardium on perfusion poses another complication, requiring an additional SPECT scan with a “pure” perfusion tracer to correct for the effect of perfusion in order to measure ΔΨ_m_^49^.

### Study limitations

For quantification of V_T_ as the ratio of tracer concentration in tissue over tracer concentration in plasma and subsequently relating it to ΔΨ_T_, the concentrations should be in secular equilibrium. For our data analysis and statistical comparison, we made the simplifying assumption that after each of the two intracoronary infusions, a new secular equilibrium was reached, allowing us to compute ΔΨ_T_ before and after each of the infusions. However, irrespective of this underlying assumption, a clear decrease in [^18^F]FTPP^+^ concentration in tissue following the DOX infusions could be observed, which is ultimately related to an underlying change in ΔΨ_T_.

Perfect secular equilibrium was not reached for some of the experiments. For those studies, the tracer concentration in tissue continued to increase slightly in the segments not exposed to DOX (non-LAD segments), resulting in an apparent hyperpolarization of the mitochondria for those segments after DOX infusion.

We assumed the [^18^F]FTPP^+^ concentration in plasma (C_pl_) to be constant starting from the first intracoronary infusion. This aspect is an additional approximation. However, importantly, when calculating δΔΨ_T,_ as δΔΨ_T_ = ΔΨ_T,after_ − ΔΨ_T,before_ and setting C_pl,before_ = C_pl,after_ for calculating V_T_ as well as the corresponding ΔΨ_T_, then δΔΨ_T_ is independent of *f*_mito_ and *f*_ECV_. Hence, our results in terms of δΔΨ_T_ were not influenced by the assumed or measured values of *f*_mito_ and *f*_ECV_.

The administered [^18^F]FTPP^+^ dose of 601.1 ± 83.9 MBq was larger than a clinically feasible dose due to imaging of over three hours with our experimental protocol. We have previously demonstrated that [^18^F]FTPP^+^ PET-based membrane potential mapping is feasible in human subjects with lower doses of ~ 400 MBq^19^.

The ΔΨ_T_ values measured with PET were not compared to ex-vivo measurements in excised tissue after animal sacrifice. However, we have previously validated the dependence of the measured imaging parameters on ΔΨ_m_ with the proton uncoupler BAM15^18^. Furthermore, we have evaluated [^18^F]FTPP^+^ PET-based quantification of ΔΨ_T_ in humans, with measured values in very good agreement with the literature^19^.

## Conclusion

[^18^F]FTPP^+^ PET mapping of cardiac membrane potential can measure acute partial depolarization of myocardial mitochondria following intracoronary DOX infusion in a large animal model. Future studies will assess the potential of this technique for early detection of DIC in a chronic setting over several cycles of chemotherapy treatment first in animals and subsequently, in a clinical study in patients.

## Supplementary Information

Supplementary Information.
