# Supplementary material for: PET imaging of mitochondrial function in acute doxorubicin-induced cardiotoxicity: a proof-of-principle study
Source: Sci Rep. 2022 Apr 12;12:6122. doi: 10.1038/s41598-022-10004-6 (PMC9005533; doi:10.1038/s41598-022-10004-6)
Supplement: Supplementary file 1 — Supplementary Information. [file 41598_2022_10004_MOESM1_ESM.pdf]

PET imaging of mitochondrial function in acute doxorubicin-induced cardiotoxicity: a proof-of-principle study - ***Supplementary Data***

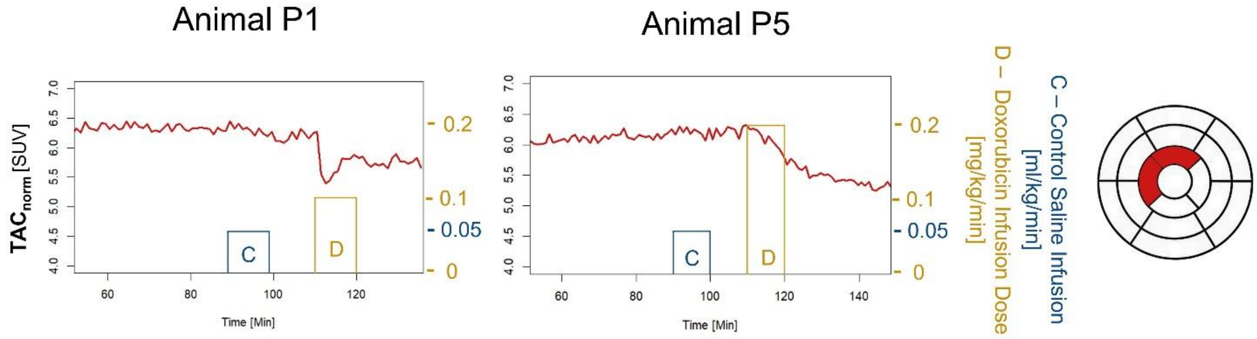

Figure S1

Normalized TACs averaged for two segments of the LAD for one example animal of group A (1 mg/kg DOX dose, left) and one example animal of group B (2 mg/kg DOX dose right). The golden line shows the rate of the DOX (D) infusion over time, the blue line indicates the volume rate of the control saline infusion (C). After the DOX infusion, the tissue tracer concentration recovered to a value close to its baseline value for the animal of group A, whereas no recovery was observed for animal B. The standard 17 segment polar map of the myocardium (right) is visualizing the location of the two segments.

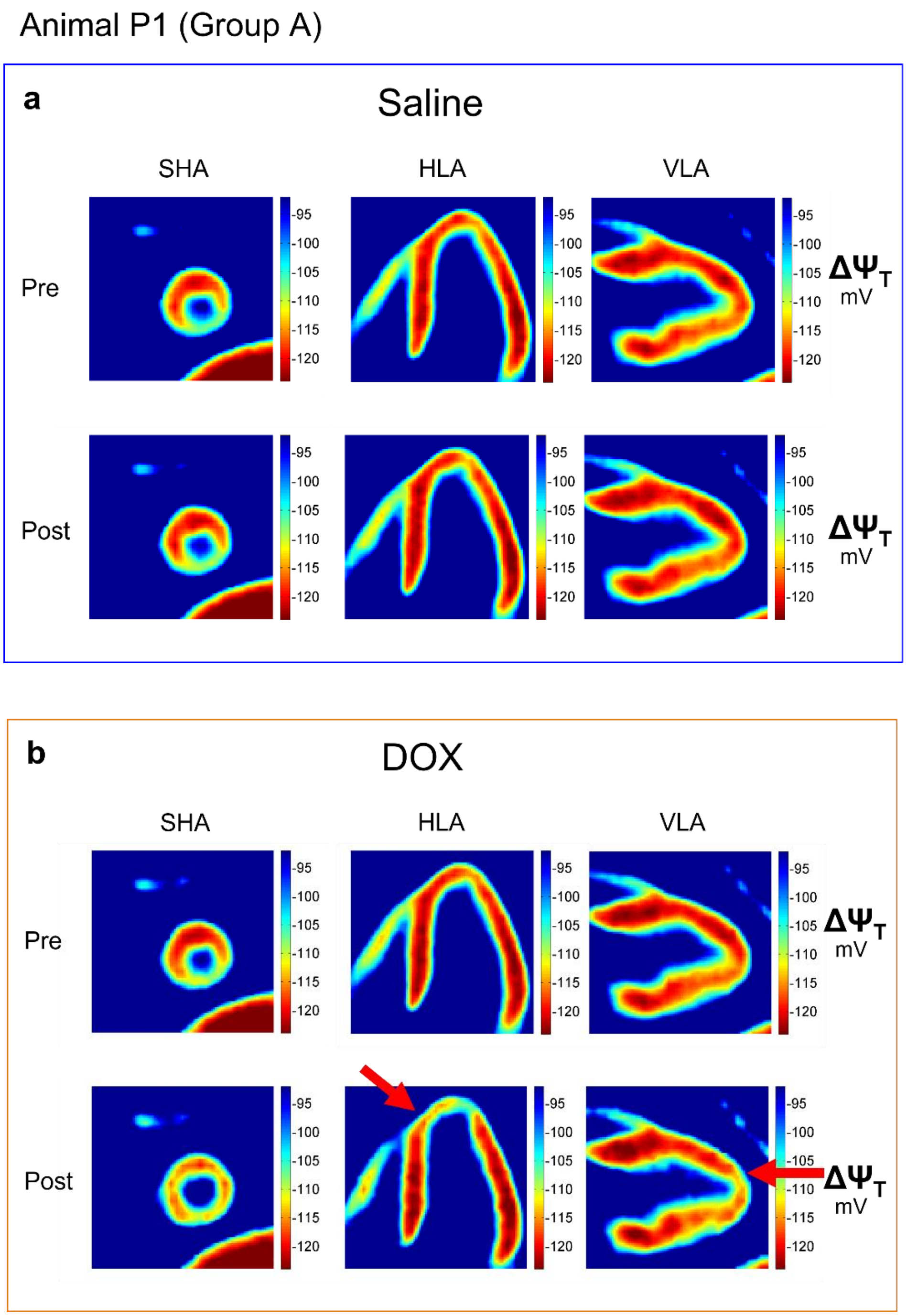

Figure S2

Parametric images of $\Delta\Psi_T$ before and after the saline (**a**) as well as the DOX infusion (**b**) for one animal of group A shown in three standard views.

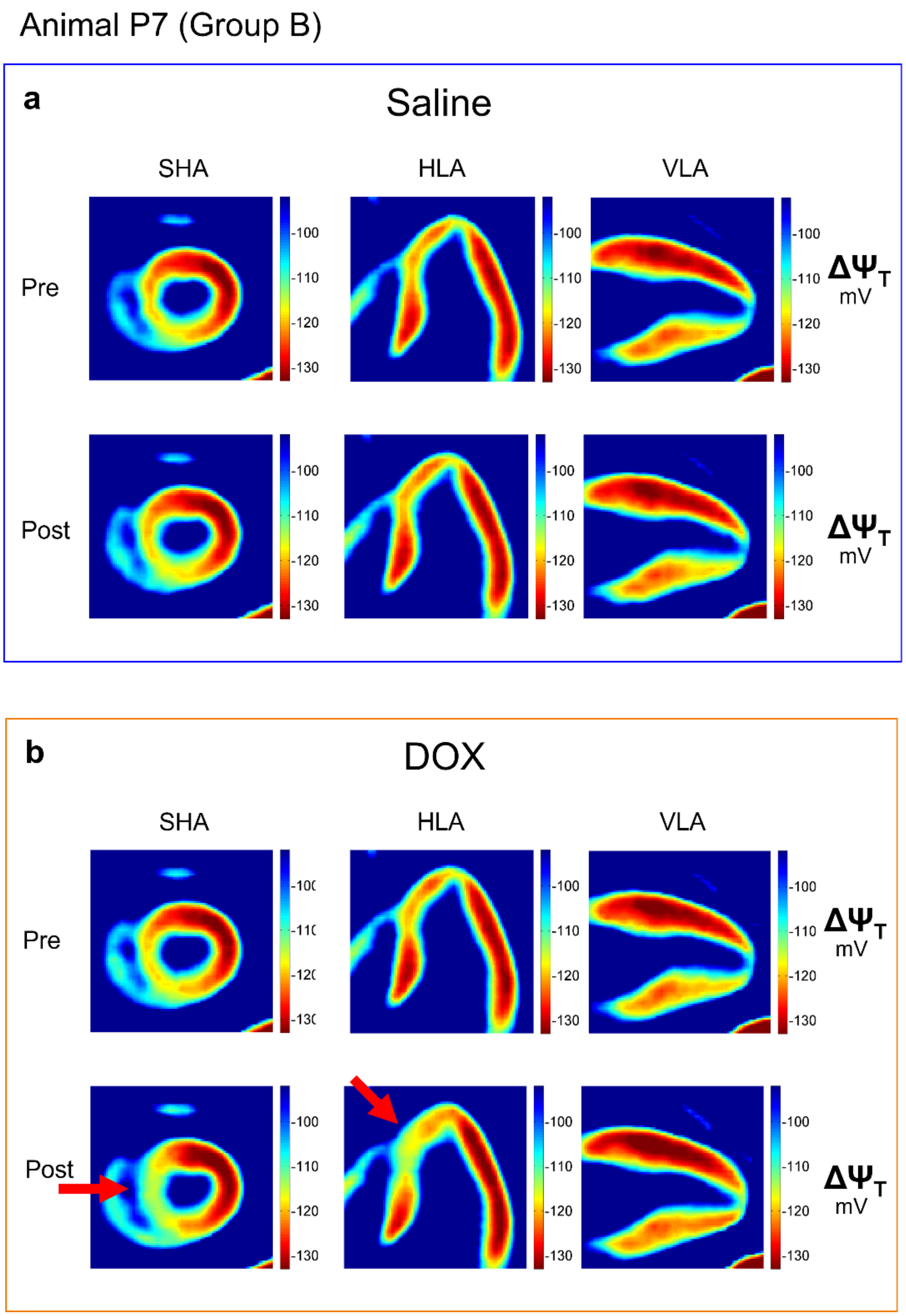

Figure S3

Parametric images of $\Delta\Psi_T$ before and after the saline (**a**) as well as the DOX infusion (**b**) for one animal of group B shown in three standard views.

Table S1 Imaging parameters and calculated f_{ECV} values for the 8 animals. For animals P3 – P8, two CT volumes were acquired before and after iodine contrast injection and used to calculate the average f_{ECV}.

PRE=pre-contrast; POST=post-contrast; Delay=time between contrast administration and post-contrast image acquisition

Animal	f_{ECV} Test	f_{ECV} Retest	Average f_{ECV}	Delay	kVp (mA) PRE test	kVp (mA) PRE retest	kVp (mA) POST test	kVp (mA) POST retest
P1	0.34581	-	0.34581	15 min	100 (200)	-	100 (200)	-
P2	0.3319	-	0.3319	15 min	100 (200)	-	100 (200)	-
P3	0.24414	0.29767	0.270905	10 min	120 (345)	120 (345)	120 (345)	120 (345)
P4	0.31991	0.28223	0.30107	10 min	120 (345)	120 (345)	120 (345)	120 (345)
P5	0.2805	0.30362	0.29206	10 min	100 (345)	100 (345)	100 (345)	100 (345)
P6	0.2914	0.28991	0.290655	10 min	100 (345)	100 (345)	100 (345)	100 (345)

P7	0.3051	0.2932	0.29915	10 min	100 (345)	100 (345)	100 (300)	100 (175)
P8	0.3221	0.334	0.32805	10 min	100 (345)	100 (345)	100 (345)	100 (345)

Table S2 For each animal, the average [^{18}F]TPP^{+} concentration in myocardium was calculated before and after the saline as well as DOX infusions by averaging the concentration starting x=0-20 minutes after the beginning of the infusion over y=5-15 minutes, indicated in the table as (x) y minutes. E.g., for animals receiving 1 mg/kg of DOX, the average [^{18}F]TPP^{+} concentration in the myocardium before the DOX infusion was determined over 10 minutes before the start of the DOX infusion, the average concentration after the DOX infusion was calculated as the mean over 5 minutes starting 5 minutes after the beginning of the DOX infusion.

	Saline Infusion	DOX Infusion	
		(1 mg/kg)	(2 mg/kg)
Before	(0) 10 minutes	(0) 10 minutes	(0) 10 minutes
After	(0) 10 minutes	(5) 5 minutes	(20) 15 minutes

Table S3 Results of the fitted linear mixed effects model. *ContrSegm* refers to the contrasts for testing hypothesis 1), *ContrDSal* for testing hypothesis 2), and *ContrD1D2* for hypothesis 3). The last two rows show the obtained values for the two tested interaction terms, assessing whether $\delta\Delta\Psi_T$ was significantly different for the saline vs. DOX infusion and DOX infusion of 1 mg/kg vs. 2 mg/kg when taking a different effect depending on the segments into account. Df=approximated degrees of freedom

	Estimate	Std. Error	Df	t-value	p-value
**(Intercept)**	-0.29419	0.1292	7.28339	-2.2775	0.0554
**ContrSegm**	4.347541	0.4169	243.3012	10.4284	<0.0001
**ContrDSal**	0.10653	0.1522	243.3012	0.6998	0.4847
**ContrD1D2**	-0.57783	0.2085	145.9596	-2.7717	0.0063
**ContrSegm:ContrDSal**	8.979525	1.0547	243.3012	8.5141	<0.0001
**ContrSegm:ContrD1D2**	3.649884	1.1186	243.3012	3.2628	0.0013
